# Death education for doctors: Introducing the perspective of death and life studies into primary care physician training

**DOI:** 10.1002/jgf2.444

**Published:** 2021-05-04

**Authors:** Daisuke Son, Makiko Iguchi, Shin‐ichi Taniguchi

**Affiliations:** ^1^ Department of Community‐based Family Medicine Faculty of Medicine Tottori University Yonago Japan; ^2^ Graduate Program in Death and Life Studies Graduate School of Applied Religious Studies Sophia University Tokyo Japan; ^3^ Department of Community‐based Family Medicine Faculty of Medicine Tottori University Yonago Japan

## Abstract

Primary care physicians need opportunities to learn about issues related to dealing with patient deaths, especially coping with emotional conflicts. Introduction of the perspective of death and life studies may have great educational significance.

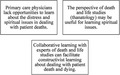

To the Editor,

One of the authors (DS) had the following experience during palliative care training at a family medicine residency. He visited a terminally ill patient and noticed a sutra by the patient's bedside. He asked, “What is that?” to which the patient replied, “I can't explain it in a few words.” Perhaps because of the sensitive nature of the patient's faith, the patient later informed the staff supervisor that the patient would refuse to see DS. The patient passed away without seeing DS again. DS was left with a feeling of bewilderment and a sense of guilt about the patient, but he could not talk to anyone about it.

Whether in a hospital ward or home care, primary care physicians often treat patients in the end‐of‐life stage, including handling patient deaths. Yet despite learning techniques for end‐of‐life care and palliative care, physicians lack opportunities to learn about issues related to spirituality or the suffering and conflicts that they face when dealing with patient deaths. Physicians may experience various kinds of suffering related to the death of a patient, but their emotions are usually covered up because of the belief that they should not have negative emotions toward patients.^1^

The perspective of death and life studies may be useful for learning how to deal with the distress and spiritual issues felt by patients near the time of their death, as well as for understanding how to manage the distress felt by physicians themselves. As a field of study, thanatology (death studies) has developed since the 1960s with the hospice movement in the UK, incorporating knowledge from philosophy, historical studies, anthropology, religious studies, etc. In Japan, the term *shiseigaku* (death and life studies) has come to be used as a counterpart to the English word thanatology, and since the 1970s, there has been an upsurge in the study of care for the dying and gatherings of those who have lost close relatives.^2^ Susumu Shimazono was a central figure in promoting and establishing death and life studies as an academic field through research projects. Shimazono states, “Death is becoming more and more distant from our daily lives, but rather than running away from it, modern people should face it anew and make sure that their attitude toward life is based on it.”^3^ In recent years, the perspective of death and life studies has become increasingly important in discussions of advanced care planning,^4^ in the hope that it would facilitate dialogue between patients and medical professionals, taking into account the Japanese view of life and death.

One approach of death and life studies is to separate the phases of “questions” and “answers” in the discourse of spirituality.^5^ For example, the spirituality of questions includes asking about matters such as “What is the ultimate meaning and purpose of life?” On the other hand, the spirituality of answers tries to offer some kind of reason, explanation, or direction to such questions. The answers change with time, culture, and context. In the practice of primary care, it is important to continue to ask the “questions” surrounding the patient, together with him/her, and to engage in a dialogue.^6^ It is possible to apply this perspective of death and life studies in reflections on the clinical practice of physicians engaged in end‐of‐life care, and we believe that it has great educational significance.

One example of a practical approach would be for experts of death and life studies to participate in reflections on end‐of‐life care during the training of residents or physicians. Collaborative reflection with experts from diverse disciplines can facilitate constructivist learning about issues for which there is no single answer. Constructivism in educational context means that learners develop conceptual understanding via the social sharing of meanings, thus emphasizes discussing one's own ideas with others. In this way, we propose providing primary care physicians in training with opportunities to learn about dealing with patient death and dying in a constructivist way through collaborative dialogue.

## CONFLICT OF INTEREST

The authors have stated explicitly that there are no conflicts of interest in connection with this article.
